# Side-to-End Lymphaticovenular Anastomosis through Temporary Lymphatic Expansion

**DOI:** 10.1371/journal.pone.0059523

**Published:** 2013-03-25

**Authors:** Takumi Yamamoto, Hidehiko Yoshimatsu, Nana Yamamoto, Mitsunaga Narushima, Takuya Iida, Isao Koshima

**Affiliations:** Department of Plastic and Reconstructive Surgery, the University of Tokyo, Tokyo, Japan; Children's Hospital Boston, United States of America

## Abstract

**Objective:**

The number of bypasses is the most important factor in lymphaticovenular anastomosis (LVA) for lymphedema treatment. Side-to-end (S-E) LVA, which can bypass bidirectional lymph flows via one anastomosis, is considered to be the most efficient bypass, but creation of lateral window to a small lymphatic vessel is technically demanding. To overcome the difficulty, we introduced S-E anastomosis through temporary lymphatic expansion (SEATTLE) procedure in S-E LVA.

**Methods:**

This was a retrospective observational study set in a teaching hospital. Forty eight lower extremity lymphedema (LEL) patients underwent LVA. S-E LVAs were performed with (SEATTLE group) or without (non-SEATTLE group) temporary lymphatic expansion. S-E LVAs were evaluated to compare anastomosis result in SEATTLE and non-SEATTLE groups.

**Results:**

S-E LVAs resulted in 44 anastomoses in SEATTLE group (n = 25) and 37 anastomoses in non-SEATTLE group (n = 23). LEL index reduction in SEATTLE group was significantly greater than that in non-SEATTLE group (16.5±14.5 vs. 10.9±11.8, *P* = 0.041). Success rate of S-E LVA in SEATTLE group was significantly higher than that in non-SEATTLE group (95.5% vs 81.1%, *P* = 0.040). Thirty seven of 44 (84.1%) lymph vessels in SEATTLE group were successfully dilated by temporary lymphatic expansion maneuver. All of 9 failed S-E LVAs used a lymphatic vessel with diameter of 0.35 mm or smaller.

**Conclusions:**

The SEATTLE procedure facilitates S-E LVA by a simple and easy maneuver. When the diameter of the lymphatic vessel is 0.35 mm or smaller even after the temporary lymphatic expansion maneuver, S-E LVA is not recommended due to relatively high failure rate.

## Introduction

Treating lymphedema refractory to conservative therapies is a great challenge [1]–[5]. With development of supermicrosurgery which allows anastomosis of vessels less than 0.5 mm in diameter, supermicrosurgical lymphaticovenular anastomosis (LVA) is becoming the treatment of choice for refractory lymphedema due to its effectiveness and minimal invasiveness [6]–[14]. Treatment efficacy of LVA has been reported to correlate with the number of lymphaticovenular anastomoses (LVAs) [10], [11], [13]. It is important to make bypasses not only with normograde distal-to-proximal lymph flow, but also with retrograde proximal-to-distal lymph flow; abnormal retrograde lymph flow always exists in lymphedema patients due to valve insufficiency of the lymphatic vessels [13].

Among various types of LVAs, side-to-end (S-E) anastomosis is considered to be the most efficient bypass, because S-E anastomosis can bypass both normograde and retrograde lymph flows with one anastomosis [11], [13]. However, S-E anastomosis is technically more challenging than end-to-end (E-E) or end-to-side (E-S), and cannot be performed by a microsurgeon with less experience of LVA. The procedure’s highest hurdle is creation of a lateral window in the wall of a small lymphatic vessel. In this study, we introduced a new method, temporary lymphatic expansion technique, to facilitate S-E LVA for lower extremity lymphedema (LEL), and evaluated its effectiveness by comparing anastomosis results between S-E LVA with and without the method.

## Methods

From July 2009 to August 2010 under the University of Tokyo Hospital ethical committee-approved protocol, 48 bilateral LEL patients (3 males and 45 females) underwent LVA surgery at The University of Tokyo Hospital, Japan. All patients included in this study received compression therapy using elastic stockings, and suffered from progressive lymphedema refractory to conservative therapy. The etiology of LELs consisted of primary lymphedema (n = 6), uterine cervical carcinoma (n = 18), uterine corpus carcinoma (n = 13), ovarian cancer (n = 6), rectal carcinoma (n = 2), prostatic carcinoma (n = 1), malignant lymphoma (n = 1), and bladder cancer (n = 1). Patients’ age ranged from 25 to 71 years old (median, 49 years old), duration of edema ranged from 8 to 216 months (median, 66 months), International Society of Lymphology stage ranged from 1 to 3 (11 in stage 1, 34 in stage 2, and 3 in stage 3) [15]. All patients gave written consents to this retrospective observational study.

### LVA Procedures

Incision sites were decided based on preoperative indocyanine green (ICG) lymphography findings, and 2-cm long incisions were usually made around the inguinal regions, the knees, and the ankles along the greater saphenous veins [16]–[21]. After detection of lymphatic vessels and venules suitable for anastomoses, lymphatic vessels were anastomosed to venules using 11-0 or 12-0 nylon [11], [13], [22]–[25]. Successful LVA is defined by confirmation of patency under an operating microscope, in which lymph-blood border movement across the site of anastomosis is observed. All S-E LVAs were performed by one surgeon (T.Y.). One week after LVA surgery, patients resumed the same conservative therapies as performed preoperatively. After January 2010, side-to-end anastomosis through temporary lymphatic expansion (SEATTLE) procedure was employed in S-E LVA.

### SEATTLE Procedures (Video S1)

**Figure 1:**
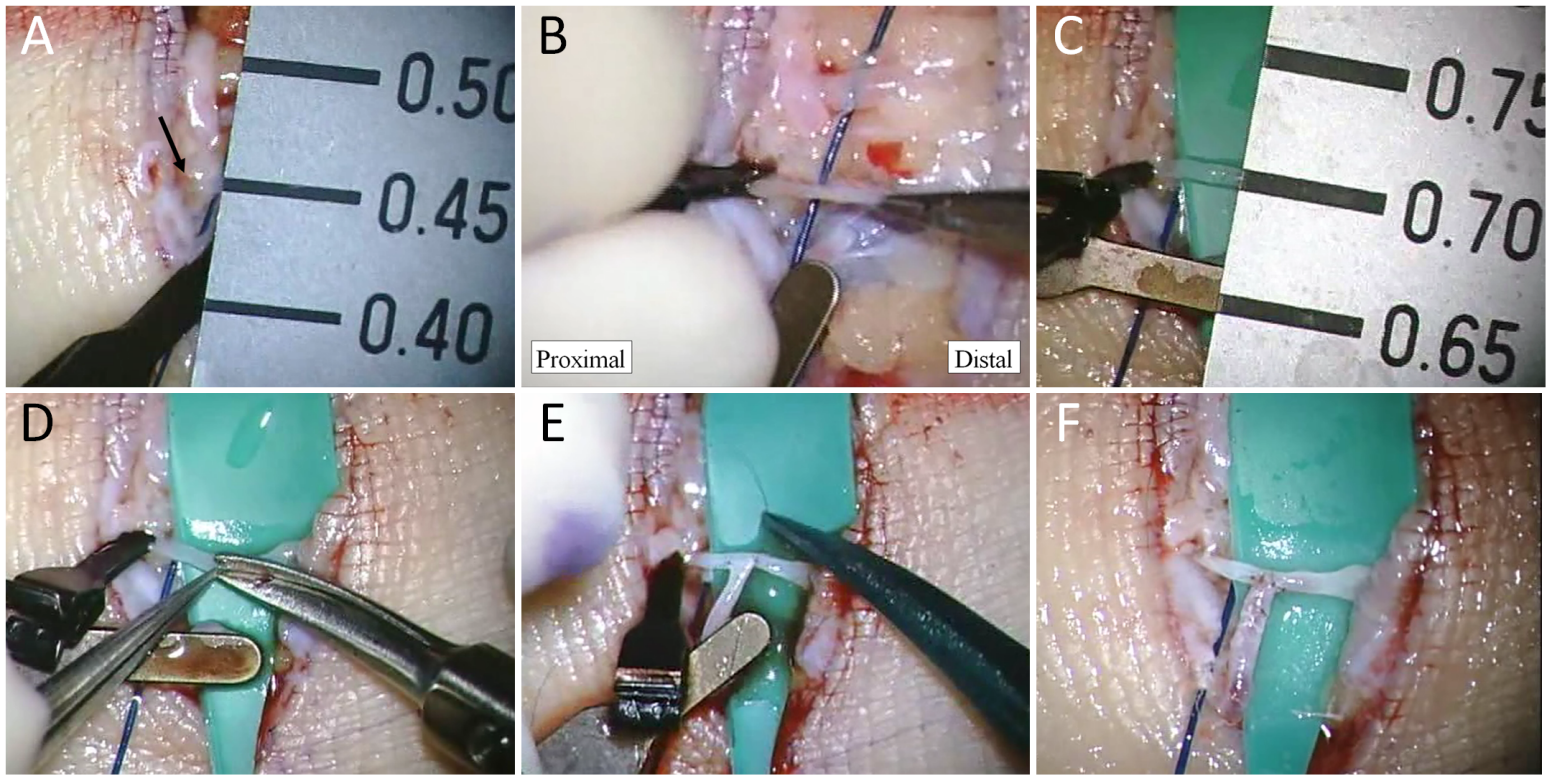
Photographs from an actual side-to-end anastomosis through temporary lymphatic expansion (SEATTLE) procedure. A lymphatic vessel with diameter of 0.45 mm (arrow) and a venule with diameter of 0.60 mm are prepared for anastomosis (A). The lymphatic vessel is clamped proximal to the anastomosis site, and then the limb distal to the anastomosis site is massaged to expand the lymphatic vessel (B). The lymphatic vessel becomes dilated to 0.70 mm in diameter via clamping and the massage, allowing easier creation of a window for S-E anastomosis, one of the most difficult procedures in S-E anastomosis (C). A window for anastomosis is created using microscissors (D). Successful creation of the window appropriate for anastomosis allows safe and easy S-E anastomosis (E). After completion of the SEATTLE procedure, patency of the anastomosis is confirmed by observing movement of lymph-blood border under an operating microscope (F). In this anastomosis, blood temporalily flowed into the lymphatic vessel (arrow heads), then the lymph-blood border moved to the venule (arrow).

Shortly before performing a S-E anastomosis, the lymphatic vessel was clamped proximal to the anastomosis site, and the limb distal to the site was manually massaged to bring lymph fluid to the region, thus expanding the lymphatic vessel (Figure 1A & 1B). The lymphatic vessel became dilated by the temporary lymphatic expansion maneuver, facilitating creation of a window for S-E anastomosis, which is the most difficult procedure in S-E LVA (Figure 1C). We used microscissors instead of fine needles to make the window, because it is easier to adjust the size of the opening (Figure 1D). Successful window creation resulted in a safe S-E anastomosis (Figure 1E). After completion of the SEATTLE procedure, patency of the anastomosis was confirmed by observing the movement of lymph-blood border under an operating microscope (Figure 1F).

### Data Collection and Statistical Analysis

Patient characteristics, operative findings, and pre- and post-operative lymphedematous volume were collected and evaluated retrospectively. Forty eight LEL patients who underwent S-E LVAs were divided into SEATTLE group and non-SEATTLE group; patients who underwent S-E LVAs with temporary lymphatic expansion maneuver were classified into SEATTLE group, and patients who underwent S-E LVAs without temporary lymphatic expansion maneuver into non-SEATTLE group. Edematous volume was evaluated preoperatively and 6 months after the operations using lower extremity lymphedema (LEL) index [26]. A summation of squares of circumferences C_1_, C_2_, C_3_, C_4_, and C_5_ (cm) divided by body mass index (BMI) is defined as the LEL index. C_1_ denotes circumference at 10 cm above the superior border of the patella, C_2_ circumference at the superior border of the patella, C_3_ circumference at 10 cm below the superior border of the patella, C_4_ circumference at the lateral malleolus, and C_5_ circumference at the dorsum of the foot. LEL index reduction after LVA surgery in SEATTLE group and non-SEATTLE group, success rate of S-E anastomosis and successful dilatation of lymph vessels by temporary lymphatic expansion maneuver in the SEATTLE group were evaluated. Chi-square test, paired Student’s *t* test, and Mann-Whitney *U* test were used appropriately for statistical analysis. The numbers after the plus-minus signs are the standard deviations. Statistical significance was defined as a *P*-value <0.05.

## Results

**Figure 2:**
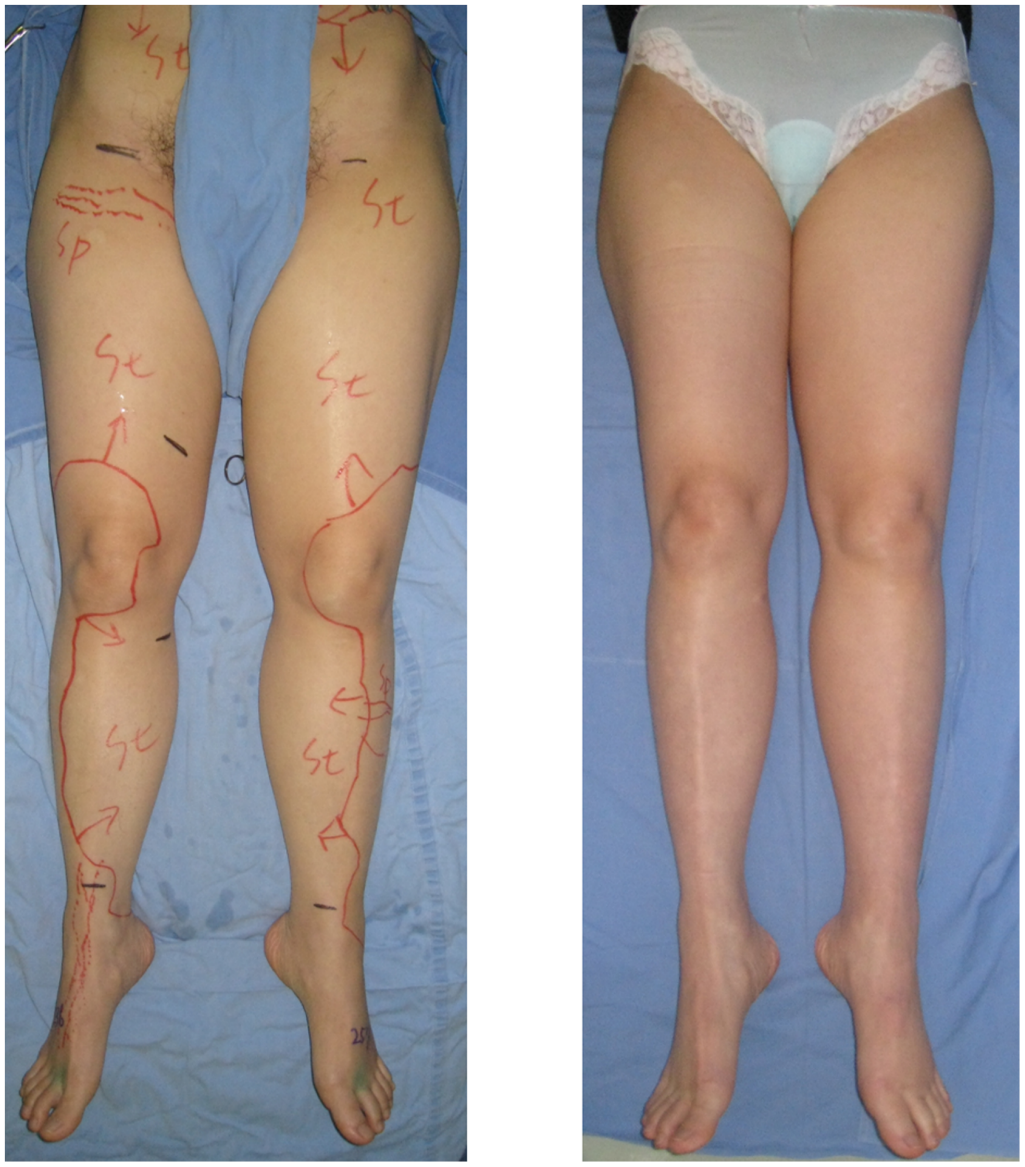
A 54-year-old female suffered from International Society of Lymphology stage 2 lower extremity lymphedema (LEL), whose LEL index of the left leg was 284 (left). Two side-to-end lymphaticovenular anastomoses were performed with temporary lymphatic expansion technique on the left leg. Six months after the operation, her left leg decreased in size, resulting in decrease of LEL index to 258 (right).

Patient demographics were shown in Table 1. S-E LVAs resulted in 37 S-E LVAs without temporary lymphatic expansion maneuver on 23 patients (non-SEATTLE group), and 44 S-E LVAs with temporary lymphatic expansion maneuver on 25 patients (SEATTLE group). In non-SEATTLE group, LEL index 6 months after LVA ranged from 190 to 288, and significantly decreased compared with preoperative LEL index (240.2±25.1 vs. 251.0±28.6, *P*<0.001). In SEATTLE group, LEL index 6 months after LVA ranged from 199 to 299, and significantly decreased compared with preoperative LEL index (236.7±23.1 vs. 253.2±29.9, *P*<0.001) (Figure 2). LEL index reduction in SEATTLE group was significantly greater that in non-SEATTLE group (16.5±14.5 vs. 10.9±11.8, *P* = 0.041). There was no statistically significant difference in LEL index reduction between primary and secondary lymphedema cases (18.2±15.9 vs. 13.2±13.1, *P* = 0.316).

**Table 1:** Patient demographics in SEATTLE and non-SEATTLE group.

			non-SEATTLE (n = 23)	SEATTLE (n = 25)
Age (years old)^a^			25–71 (48)	26–70 (52)
Gender		female	21 (91.3%)	24 (96.0%)
		male	2 (8.7%)	1 (4.0%)
Duration of edema (months)^a^			8–192 (60)	12–216 (72)
ISL stage		stage 1	6 (26.1%)	5 (20.0%)
		stage 2	16 (69.6%)	18 (72.0%)
		stage 3	1 (4.3%)	2 (8.0%)
Etiology of lymphedema	primary		4 (17.4%)	2 (8.0%)
	secondary	uterine cervical carcinoma	8 (34.8%)	10 (40.0%)
		uterine corpus carcinoma	5 (21.7%)	8 (32.0%)
		ovarian cancer	4 (17.4%)	2 (8.0%)
		other cancers^b^	2 (8.7%)	3 (12.0%)

SEATTLE, side-to-end anastomosis through temporary lymphatic expansion. ISL, International Society of Lymphology. Data are counts (percentages) otherwise indicated.

a Data are ranges (medians).

b Other cancers include rectal carcinoma, prostatic carcinoma, malignant lymphoma, and bladder cancer.

Comparison of intraoperative findings between non-SEATTLE and SEATTLE groups revealed significant differences in diameter of lymph vessel after expansion maneuver (0.492±0.177 vs. 0.602±0.230, *P* = 0.017) and success rate of S-E anastomosis (81.1% vs. 95.5%, *P* = 0.040) (Table 2). Thirty-seven of 44 (84.1%) lymph vessels in SEATTLE group were successfully dilated by temporary lymphatic expansion maneuver, and diameter of lymph vessels after expansion were significantly larger than those before the maneuver (0.602±0.230 vs. 0.481±0.195, *P*<0.001). No obvious adverse effect was observed due to temporary lymphatic expansion maneuver, such as obstruction or stenosis of a lymphatic vessel at the site of clamping. Nine of 81 S-E LVAs resulted in anastomosis failure; 7 of which were in non-SEATTLE group, and 2 of which were in SEATTLE group (Table 3). All of 9 failed S-E LVAs used a lymphatic vessel with diameter of 0.35 mm or smaller, and were re-anastomosed in an E-E or E-S fashion; 6 of which were successfully re-anastomosed, but 3 of which resulted in re-anastomosis failure. The main reason for the re-anastomosis failure was the shortness of vessels’ length after removal of the failed anastomosis sites.

**Table 2:** Comparison of intraoperative findings between non-SEATTLE and SEATTLE groups.

	non-SEATTLE (n = 37)	SEATTLE (n = 44)	
	Mean ± SD	Mean ± SD	P-value
Elapsed time (minute) for anastomosis	10.6±3.5	10.3±3.8^a^	0.739
Diameter of lymphatic vessel before expansion maneuver (mm)	0.492±0.177	0.481±0.195	0.787
Diameter of lymphatic vessel after expansion maneuver (mm)	−	0.602±0.230	0.017^b^
Diameter of venule (mm)	0.461±0.223	0.443±0.244	0.735
Success rate^c^	30/37 (81.1%)	42/44 (95.5%)	0.040

SEATTLE, side-to-end anastomosis through temporary lymphatic expansion; SD, standard deviation.

a Including time for clamping and manual massage.

b Compared with diameter of lymph vessel before expansion maneuver in non-SEATTLE group.

c Data are counts (percentages).

**Table 3:** Failure in 9 of 81 S-E anastomoses.

No.	Procedure	Diameter of lymphatic vessel (mm)	Diameter of venule (mm)	Salvage anastomosis	Result of salvage
1	non-SEATTLE	0.20	0.30	E-E	Failure
2	non-SEATTLE	0.25	0.35	E-E	Failure
3	non-SEATTLE	0.25	0.35	E-E and E-S	Success
4	non-SEATTLE	0.30	0.50	E-E and E-S	Success
5	non-SEATTLE	0.30	0.20	E-E	Success
6	non-SEATTLE	0.35	0.35	E-E and E-S	Success
7	non-SEATTLE	0.35	0.35	E-E and E-S	Success
8	SEATTLE	0.25 (0.25)^a^	0.40	E-E	Success
9	SEATTLE	0.20 (0.35)^a^	0.15	E-E	Failure

SEATTLE, side-to-end anastomosis through temporary lymphatic expansion; S-E, side-to-end anastomosis; E-E, end-to-end anastomosis; E-S, end-to-side anastomosis.

a Diameter of lymphatic vessel before (after) temporary lymphatic expansion maneuver.

## Discussion

This study revealed that SEATTLE procedure improved success rate of S-E LVA, and that volume reduction in SEATTLE group was significantly greater than that in non-SEATTLE group. SEATTLE procedure successfully dilates less-sclerotic lymphatic vessels by a simple and easy maneuver, which only requires clamping and manual massage without harmful effect on lymphatic vessels. Although dilatation of a lymphatic vessel is temporary, this temporary lymphatic expansion maneuver dilates the vessel by about 0.12 mm, making creation of a lateral window in a lymphatic vessel much easier.

LVA requires supermicrosurgical technique, anastomosis of vessels with diameter of around 0.5 mm. To facilitate technically demanding supermicrosurgical anastomosis, several stenting methods have been reported; a nylon thread is inserted into a lymphatic vessel to keep the vessel’s lumen open [11], [13], [22]. Using stenting methods, a surgeon can anastomose lymphatic vessels with more ease and confidence. Although several technical refinements in anastomosis have been reported, no technique for lymphotomy in S-E LVA has been reported. For microsurgeons with experience of LVA surgery, anastomosing lymphatic vessels is not difficult, but lymphotomy, creating a window on a lymphatic vessel is challenging. It is difficult to create a window in a small lymphatic vessel, so a larger lymphatic vessel is favorable to be used in a S-E LVA [11], [13]. Successful window creation is the key to establishment of a successful S-E LVA. If the window is too large relative to the diameter of the lymphatic vessel, the disposition of the vessels tends to be tortuous, resulting in poor patency. Higher success rate of S-E LVA in SEATTLE group than that in non-SEATTLE group suggests that the temporary expansion maneuver plays an important role in improving anastomosis result of S-E LVA.

S-E LVA is the most recommended type of anastomoses and should be considered as the first choice whenever possible, because it can make bidirectional lymph flow bypasses in one anastomosis, preserve a native recipient lymph flow, and show less venous backflow after anastomosis [10], [11], [13]. However, S-E LVA is technically difficult, especially when diameter of a lymphatic vessel is 0.35 mm or less. As shown in Table 3, all failed S-E LVAs used lymphatic vessels with diameter of 0.35 mm or less. Failure in primary S-E LVA makes secondary end-to-end (E-E) or end-to-side (E-S) anastomosis more difficult than the primary anastomosis. This is because the lymphatic vessel and the venule become shorter after removal of the failed anastomosis site.

We recommend following strategy in selecting anastomosis type. First, temporary expansion maneuver should be performed. Then, a S-E LVA should be employed when diameter of a lymphatic vessel is larger than 0.35 mm; otherwise an E-E or E-S LVA should be selected.

Major limitations of this study are that this study was a retrospective observational study, and that the effectiveness of our technique is not supported by long-term results or anastomosis patency. Our previous reports revealed short-term and long-term effectiveness of LVA surgery using various types of anastomoses [7]–[9], [12], [13]. Since volume reduction after LVA surgery results from bypassing congested lymph into venous circulation, anastomosis patency would be the most important prognostic factor, and should be evaluated postoperatively. Although this study revealed significant positive effect on volume reduction by SEATTLE procedure compared with S-E LVA without temporary lymphatic expansion, further prospective controlled studies are needed to clarify long-term effectiveness of the procedure evidenced with long-term anastomosis patency.

### Conclusions

The SEATTLE procedure facilitates successful S-E LVA by a simple and easy maneuver. To avoid anastomosis failure, S-E LVA should not be employed when diameter of the lymphatic vessel is 0.35 mm or smaller after temporary lymphatic expansion maneuver.

## Supporting Information

Video S1 **A lymphatic vessel with diameter of 0.45 mm and a venule with diameter of 0.60 mm are prepared for anastomosis.** The lymphatic vessel is clamped proximal to the anastomosis site, and the limb distal to the anastomosis site is massaged to expand the lymphatic vessel. The lymphatic vessel becomes dilated to 0.70 mm in diameter via clamping and the massage, allowing easier creation of a window for S-E anastomosis, one of the most difficult procedures in S-E anastomosis. A window for anastomosis is created using microscissors. Successful creation of the window appropriate for anastomosis allows safe and easy S-E anastomosis. After completion of the SEATTLE procedure, patency of the anastomosis is confirmed by observing a movement of lymph-blood border under an operating microscope. In this anastomosis, blood temporalily flowed into the lymphatic vessel, then the lymph-blood border moved to the venule.
(WMV)
